# Big Data Offers Novel Insights for Oncolytic Virus Immunotherapy

**DOI:** 10.3390/v8020045

**Published:** 2016-02-05

**Authors:** Stephanie L. Swift, David F. Stojdl

**Affiliations:** 1 Children’s Hospital of Eastern Ontario Research Institute, 401 Smyth Road, Ottawa, ON K1H 8L1, Canada; stephanies@arc.cheo.ca; 2 Department of Biology, Microbiology and Immunology, University of Ottawa, Ottawa, ON K1N 6N5, Canada; 3 Department of Pediatrics, University of Ottawa, Ottawa, ON K1N 6N5, Canada

**Keywords:** oncolytic, virus, immunotherapy, immunology, immune, screen, large-scale

## Abstract

Large-scale assays, such as microarrays, next-generation sequencing and various “omics” technologies, have explored multiple aspects of the immune response following virus infection, often from a public health perspective. Yet a lack of similar data exists for monitoring immune engagement during oncolytic virus immunotherapy (OVIT) in the cancer setting. Tracking immune signatures at the tumour site can create a snapshot or longitudinally analyse immune cell activation, infiltration and functionality within global populations or individual cells. Mapping immune changes over the course of oncolytic biotherapy—from initial infection to tumour stabilisation/regression through to long-term cure or escape/relapse—has the potential to generate important therapeutic insights around virus-host interactions. Further, correlating such immune signatures with specific tumour outcomes has significant value for guiding the development of novel oncolytic virus immunotherapy strategies. Here, we provide insights for OVIT from large-scale analyses of immune populations in the infection, vaccination and immunotherapy setting. We analyse several approaches to manipulating immune engagement during OVIT. We further explore immunocentric changes in the tumour tissue following immunotherapy, and compile several immune signatures of therapeutic success. Ultimately, we highlight clinically relevant large-scale approaches with the potential to strengthen future oncolytic strategies to optimally engage the immune system.

## 1. Introduction

Oncolytic viruses (OVs) represent a bioactive class of cancer immunotherapies whose anti-tumour benefit arises not only from the preferential infection and direct lysis of tumour cells [1,2,3] and mediation of vascular occlusion [4,5], but also from the stimulation of immune responses, the establishment of an inflammatory, immunogenic tumour milieu, and the modulation of an immunosuppressive tumour microenvironment [6,7,8,9,10,11,12,13,14]. Indeed, engagement of the immune response is now considered critical for OV efficacy, resulting in the redefinition of this approach as oncolytic virus immunotherapy (OVIT) rather than the more traditional oncolytic virotherapy (OVT) [9,14,15,16,17].

**Figure 1:**
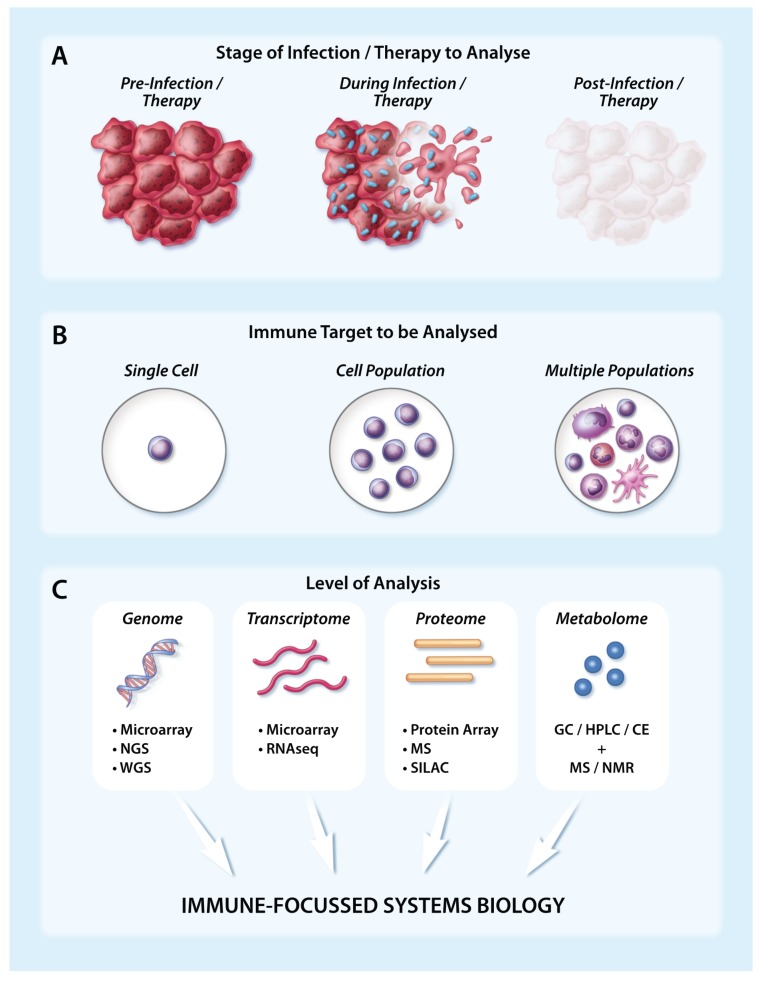
Analysing immune targets using large-scale technologies in the tumour setting following infection/therapy. (**A**) The immune response to infection or immunotherapy in the tumour setting can be captured at multiple different timepoints, including prior to infection/therapy (left), during infection/therapy as cancer cells begin to die (middle), and following infection/therapy when the tumour has been ablated or reduced in bulkiness (right); (**B**) Large-scale immune analysis can be performed at the level of a single immune cell, an immune cell population or multiple immune cell populations; (**C**) Several key large-scale techniques can capture genomic, transcriptomic, proteomic or metabolomic changes at the immune level. When assays have been appropriately replicated and validated, matched data generated at the DNA, RNA and protein level can subsequently be integrated to achieve a systems biology approach. Abbreviations: CE = Capillary Electrophoresis, GC = Gas Chromatography, HPLC = High Performance Liquid Chromatography, MS = Mass Spectrometry, NGS = Next-Generation Sequencing, NMR = Nuclear Magnetic Resonance, RNASeq = RNA Sequencing, SILAC = stable isotope labelling by amino acids in cell culture, WGS = Whole Genome Sequencing.

Infection with a replication-competent virus induces a broad number of highly dynamic and interactive immune changes. Almost all approaches to the large-scale analysis (*i.e.*, the global interrogation of complex biological systems using massively parallel molecular and computational techniques) of host-virus interactions in the infectious disease setting have focussed on mining data with the potential to suppress viral activity. However, generating insights for OVIT requires a very different focus, since the goal is typically to promote pathogenic virus-mediated host tissue destruction at the tumour site while simultaneously activating immune responses that can mediate both tumour cell death and virus control. Few studies have attempted to delineate how multiple malignancy-associated immune cell populations react following OV infection on the large-scale.

A variety of large-scale techniques can capture and map comprehensive networks of immune-related actions and reactions at different stages of infection/therapy at the level of the genome, transcriptome, proteome and/or metabolome to assess the contribution and functional status of distinct innate and adaptive immune cells (Figure 1). This can be achieved at the population level using broadly defined signatures or within individual cells (Figure 1). Large-scale assays, including microarray, whole genome sequencing (WGS), next-generation sequencing (NGS) and various “omics” technologies (Figure 1), offer unprecedented data-rich insights into the cellular changes that occur during infection of both healthy animals and those with malignant disease. Rigorous replication and validation steps are key to ensuring that these insights represent robust observations, and to ultimately facilitate the translation of pre-clinical findings into clinical interventions. Several large-scale host-virus interactome networks are publicly available for different viral species, including OVs like vesicular stomatitis virus (VSV) and vaccinia virus (VV) [18]. Further, with the comprehensive networks created through studies such as the immunological genome project, which has characterised the transcriptomes of ~270 mouse leucocyte subsets [19,20], and the human proteome map, which has proteomically profiled at least 6 human immune cell populations [21], tracking the localisation and contribution of different immune cells following infection has become feasible. However, it is important to note that many large-scale technologies remain relatively expensive and, as a consequence, are not yet in widespread use despite their clear potential for achieving novel, therapeutically-relevant insights.

Characterising the network of immune interactions that lead to long-term therapeutic efficacy or tumour relapse following OVIT has the potential to deliver new insights that may strengthen therapeutic outcomes. In this review, we analyse the use of large-scale data to characterise host immune responses to virus infection in both the healthy and tumour-bearing setting, incorporating evidence from infection, vaccination and immunotherapy studies to generate broad insights for OVIT. We explore both *in vitro* and *in vivo* studies across mouse, human and clinical systems that have generated large volumes of integrated data that extend from the molecule and the cell to the tissue and the whole body.

## 2. Viruses *vs.* Host Immunity

Following virus infection in a healthy host, target cells respond by activating multiple pathways designed to counter pathogenic activity. Although different inoculation routes have an impact on the subsequent mode of immune system activation [22], generally, virus molecular signals (pathogen-associated molecular patterns; PAMPs) are detected by membrane-associated and intracellular host pattern recognition receptors (PRRs) [23]. This initiates signalling cascades that activate transcription factors (TFs), such as NFκB [24], to induce the expression of type I interferons (IFNs), cytokines and chemokines that subsequently stimulate the expression of hundreds of interferon-stimulated genes (ISGs) [25]. These ISGs activate local immune populations, such as dendritic cells (DCs) and macrophages, which maintain gradients of inflammatory molecules and chemokines to attract other innate cells. Neutrophils are typically one of the first responders [26], with subsequent infiltration of natural killer (NK) cells, DCs and granulocytes. As infection proceeds, antigen-presenting cells (APCs) present antigens acquired through direct infection (direct presentation) or phagocytosis (cross-presentation) to recruit adaptive immune populations, including B and T cells, to solidify viral control.

In the tumour-bearing host, the immune response typically undergoes several functional alterations at both the local and global scale. Subversion of the immune response is considered one of the hallmarks of cancer cells [27], and one of the key malignant changes that enables oncolytic viruses to preferentially replicate at the tumour site [28]. Approximately 80% of cancer cell lines are defective in their IFN response [29], although in a heterogeneous tumour composed of multiple sub-clones across a genetic spectrum, there is typically a range of IFN responsiveness. This can lead to a lack of resultant immune responses downstream of IFN induction. For example, tumour cells may be able to produce type I IFNs, but remain incapable of responding to them [30]. The establishment of suppressive immune populations, including M2 macrophages, regulatory T cells (Tregs) and myeloid-derived suppressor cells (MDSCs), within the tumour microenvironment also acts to dampen local immune cell activity. Large-scale assays can capture both anti-tumour and pro-tumour immune cells (or their products) that are capable of modulating immune activity at the tumour site.

## 3. Using Large-Scale Approaches to Capture Cellular Immune Responses

***Neutrophils*:** Transcriptional signatures have uncovered the negative contributions of various inflammatory and cellular immune compartments that impact outcomes in an infected host. For example, the pathological role of pro-inflammatory and cell death signals from either haematopoietic or non-haematopoietic populations in the lung following influenza virus infection has been documented using transcriptomic analysis of broad biological process modules [31,32]. Based on transcriptional signatures, this research uncovered a pathological role for neutrophils in the lungs of mice infected with lethal but not sub-lethal strains of influenza A virus (IAV) [31]. This guided therapeutic interventions, since attenuation (but not ablation) of neutrophils during IAV infection increased survival [31]. Capturing the expression of *Ly6G*, a key neutrophil marker, as a top differentially expressed gene in microarray transcriptome data has also helped to identify neutrophils as negative outcome contributors in other models of disease [33]. Linking such cellular insights to negative outcomes can drive the design of new immunotherapies that release chemoattractants to selectively recruit T cells while minimising neutrophil and inflammatory monocyte recruitment [34].

***Dendritic Cells*:** Banchereau *et al.* explored the transcriptomic response of DCs following IAV infection. Using an *in vitro* system, human monocytes were matured with GM-CSF together with either IL-4 or IFNα to generate either immature or activated DCs, respectively. Each DC population was exposed to IAV (H1N1) across multiple timepoints up to 24 h. Gene expression array analysis and subsequent tree clustering demonstrated that each DC population reacted differently to infection. DCs matured with IFNα overexpressed transcripts associated with type I IFNs, inflammation and anti-viral modules, while DCs matured with IL-4 expressed high levels of histone, ribosome and type III IFN transcripts [35]. This suggests that it may be important to validate responses to virus-based immunotherapies in specific DC subsets rather than across broad DC populations, since distinct differences can be observed in responses to virus infection.

***T cells*:** T cell infiltration at the tumour site correlates with improved patient prognosis across multiple cancer types [36,37,38], and a lack of *in situ* T cell activity can prevent tumour clearance. T cells are also critically required for the anti-tumour efficacy of most, if not all, oncolytic viruses, including VSV, herpes simplex virus (HSV), poliovirus, Newcastle disease virus (NDV) and Sindbis virus (SINV) [9,14,15,16,17]. Since IFNγ is rapidly induced following T cell receptor (TCR) stimulation, it can be used as a surrogate marker to track T cell infiltration into the tumour [39].

Several studies have tracked the functionality and/or activation status of T cells following vaccination or infection. For example, following therapeutic vaccination with a replication-incompetent recombinant human adenovirus serotype 5 (rHuAd5) vector expressing a relevant tumour-associated antigen (TAA) in tumour-bearing animals, microarray analysis uncovered the progressive appearance of immunosuppressive genes in the tumour microenvironment, including *Pdcd1* (PD-1), *HAVCR2* (TIM-3) and *LAG3* (CD223). In this setting, animals ultimately did not achieve durable tumour cures [40]. Similarly, infection of non-tumour-bearing animals with VSV expressing ovalbumin (OVA) (VSV-OVA) was associated with greater expression of suppressive *Ctla4* and *Pdcd1* on OVA-specific effector and memory CD8+ T cell populations compared to the same cells responding to infection with *Listeria monocytogenes* expressing OVA (Lm-OVA) [41]. *Slfn5*, which can repress T cell proliferation, was also upregulated in naive and late memory CD8+ T cells following VSV-OVA infection [41]. All animals ultimately recovered from infection despite this evidence of CD8+ T cell immune suppression. Collectively, these studies imply that core genes responsible for modulating T cell activity can be upregulated (albeit to different degrees) by multiple independent infectious agents, suggesting that the efficacy of most microbe-based immunotherapies may benefit from modulating these key T cell targets [41].

Capturing the expression profile of core T cell genes can be used to predict the memory precursor potential of CD8+ T cells after infection. This has important implications for promoting the establishment of long-lived anti-tumour CD8+ T cells following OVIT that may be able to protect against tumour relapse. Taking a reverse engineering approach by interrogating CD8+ T cell transcriptomic data generated from the Immunological Genome Project, Best *et al.* predicted that the transcriptional regulators, *Id2*, *Tbx21* (Tbet) and *Prdm1*, were associated with the formation of short-term effector-memory T cells, while *Tcf7* was associated with the formation of long-lived memory T cell populations [41]. *RORa* was also identified as a potential regulator of effector CD8+ T cells. Intriguingly, a single effector molecule could regulate activity across multiple memory populations. For example, cluster analysis within the CD8+ T cell compartment suggested that *Tbx21* could promote effector and effector-memory differentiation, but repressed the formation of naive and late-memory cells. The exploration of such complex networks has the potential to provide important insights for OVIT strategies where the development of T cells with particular phenotypes has therapeutic relevance.

Martin *et al.* further explored temporal gene expression changes as CD8+ T cells progressed to memory following infection. The transcriptomes of both early and late memory CD8+ T cells were analysed following infection with lymphocytic choriomeningitis virus (LCMV; Armstrong) [42]. Multiple genes involved in the cell cycle, mitochondrial function and ribosome biogenesis were upregulated in late *vs.* early memory phases. During secondary expansion, these gene changes provided a metabolic advantage through preferential energy generation via oxidative phosphorylation, thus conferring greater proliferative potential to late memory cells [42]. This is an important insight for oncolytic strategies that employ a prime/boost vaccination approach. In the prime/boost scenario, two heterologous vectors expressing a shared TAA target are administered sequentially: the first vector primes the system against the TAA target to establish a baseline of TAA-specific CD8+ T cells (primary expansion), and the second vector (typically an oncolytic virus) is applied several days or weeks later to boost these CD8+ T cell frequencies to large, curative levels (secondary expansion) [43,44]. The data generated by Martin *et al.* would predict that a greater lag period between the prime and boost vaccination would allow memory CD8+ T cells to respond more vigorously to the subsequent boost. However, such timing issues would have to be tempered with considerations that aggressive, fast-growing tumours have a limited window to achieve therapeutically relevant responses.

Finally, taking an active reverse engineering approach, Zhou *et al.* transfected short hairpin RNA (shRNA) pools into transgenic OVA-specific OT-I CD8+ T cells, infused these cells into B16.F10-OVA tumour-bearing mice, and analysed which T cell clones were enriched in tumour tissues [45]. These shRNA libraries were designed to specifically target 255 genes involved in T cell dysfunction, and 1307 genes encoding kinases/phosphatases. T cells with shRNA knockdown of *Ppp2r2d*, *Eif2ak3* and *Smad2* were highly enriched, suggesting these genes—or their downstream pathways—may contribute towards T cell dysfunction in the tumour setting. Indeed, knockdown of *Ppp2r2d* allowed greater T cell proliferation in the presence of antigen at the tumour site, and improved survival despite microenvironment inhibition and a lack of co-stimulatory support. Such approaches have potential relevance for strategies that combine OVIT with adoptive cell therapy (ACT) [46,47,48], since T cells could be drugged during dual immunotherapy to magnify anti-tumour activity.

***Tregs*:** Activated Tregs are typically present in the tumour microenvironment, where they promote tumour success by suppressing the activity of infiltrating tumour-reactive CD8+ T cells [49]. Understanding more about how immunosuppressive Treg populations signal following immune activation has the potential to inform targeted interventions designed to quench Treg activity during OVIT. Albert *et al.* simultaneously compared microRNA (miRNA) and mRNA profiles in resting or activated Tregs [50]. After integrating both miRNA and mRNA profiles, 17 unique pathways were enriched in activated Tregs compared to resting Tregs. This included several immune-specific pathways, such as TCR-, TLR- and mTOR-mediated pathways. Interestingly, several non-immune molecules were also enriched, including *FAS* and *Casp8*, indicating that activated Tregs were likely sensitive to FAS-mediated apoptosis. Such pathways may represent potential targets for disruption to minimise the contribution of Tregs to the immunosuppressive phenotype.

***M1/M2 Macrophages*:** Typically, M1 macrophages are considered pro-inflammatory and immunostimulatory (and therefore potentially beneficial during OVIT), while M2 macrophages are considered anti-inflammatory and immunosuppressive [51]. Using an unbiased high-throughput integrated approach, Jha *et al.* compared unpolarised, pro-inflammatory and anti-inflammatory macrophage populations to characterise the metabolo-transcriptional networks that contributed to the polarisation of M1/M2 macrophages [52]. M2 polarisation was critically dependent on both the UDP-GlcNAc synthesis pathway and glutamine metabolism, such that transient glutamine depletion negatively impacted M2 activation. Conversely, M1 polarisation was critically dependent on a break in the TCA cycle at *Idh*. Such approaches may have the potential to inform new strategies to promote M1 polarisation while reducing M2 activity at the tumour site during OVIT.

## 4. Using Large-Scale Approaches to Track Global Immune Changes

The distinct infiltrating phases of a cellular anti-viral response, including B, T and NK cells, can be captured by analysing the genomic, transcriptomic, proteomic and/or metabolomic signatures of an infected tissue. Indeed, key probe sets and modules can be used to define distinct immune cell populations to support systems-scale analysis for translational research [53]. Several elegant studies have been performed that temporally track global responses following *in vivo* vaccination and/or infection.

Multiple studies have tracked the global transcriptome in mouse lungs for 60 days following infection with a non-lethal strain of IAV (H1N1; PR8) to capture different phases of the immune response [54,55]. At early timepoints, most upregulated genes were associated with cytokines/chemokines and IFN-responsive genes, in addition to a strong NK cell-related signature. At intermediate timepoints, genes involved in T cell activation and apoptosis were over-represented. Subsequently, FcγR-mediated phagocytosis pathways representative of B cell activation signatures were captured in transcriptomic data. Viral loads began to decline as these signatures of adaptive T and B cell populations began to dominate the transcriptome. While FcγR-mediated phagocytosis and complement/coagulation processes remained active until day 40 [55], final responses at day 60 post-infection continued to capture many processes involved in wound healing, organ remodelling and differentiation, suggesting that tissue repair pathways remained active long after virus was cleared. Indeed, more than 500 genes remained differentially expressed at this final timepoint [54].

Different clinical phases of infection can also be tracked using key sets of enriched genes that characterise immune population(s) of interest. Vanwolleghem *et al.* compared patient whole blood transcriptomes and cytokinomes at different stages of chronic hepatitis B virus (HBV) infection, including immune tolerant, immune active, inactive carrier or negative for HBV antigen [56]. Multiplex cytokine analysis demonstrated that MCP-1, IP-10, MIP-1β and IL-12p40 varied between clinical phases, suggesting a significant modulation of the monocyte-macrophage compartment during HBV infection [56]. Sixty four unique genes were upregulated in active *vs.* tolerogenic clinical phases, with over half of those representing immunoglobulin-encoding genes involved in B cell function. T cell modules were also upregulated in patients within the immune-active phase of HBV infection. Interestingly, NK cell modules were highly upregulated during clinical phases that were associated with biochemical liver damage. A similar systems biology analysis could generate an immune fingerprint following the clinical phases of OV therapy to track relative immune cell contributions during active infection through to viral clearance, during tissue repair following tumour ablation, and, potentially, during tumour relapse.

## 5. Using Large-Scale Approaches to Enhance Immune Engagement during Oncolytic Virotherapy

When single-target therapies are unable to impact a genomically flexible tumour, combination therapies are critical to achieving long-term control. OVIT can be combined with one or multiple immunotherapeutic partners to improve anti-tumour efficacy. Thus far, most approaches have focussed on identifying therapeutic partners that can additively or synergistically enhance virus-specific parameters, such as immune evasion, cytotoxicity, lytic capacity and virus spread. Indeed, our lab published the first large-scale RNAi screen designed to identify host cell targets capable of modulating tumour oncolysis during OV infection [57]. Only a relatively small number of studies have used large-scale approaches to enhance immune activation in the context of standalone or combination OVIT.

Al-Yahya *et al.* identified several modulators of the IFN response in the context of an OV infection [58]. In this system, Huh-7 cells were transfected with an interferon-sensitive response element fused to EGFP, then treated with one of >200 individual cytokines/chemokines together with IFNα for 16 h, followed by infection with VSV (MOI = 1). EGFP expression was used as a readout of an activated IFN response. Both betacellulin (BTC) and IL-17F were identified as novel modulators of the IFNα response to VSV, since they enhanced the ability of IFNα to protect cells. This suggested that strategies to knock out BTC or IL-17F function during oncolytic rhabdoviral immunotherapy may have potential value for enhancing cell death.

*In vivo* siRNA screens have also uncovered important innate immune host factors that contribute to defense against virus infection. Varble *et al.* created a library of oncolytic SINVs encoding short-interfering RNAs (siRNAs) targeting 10,000 murine open reading frames (ORFs) [59]. In the context of an *in vivo* infection, virus clones expressing replication-enhancing siRNAs outpaced inferior clones to dominate the circulating viral population. Clones targeting two host genes—Zinc finger X-chromosomal protein (*ZfX*) or MAX gene-associated protein (*Mga*)—represented the most abundant viral populations across parallel screens. Further analysis suggested that both genes modified the anti-viral response following SINV infection, as ISG expression was reduced following infection [59]. Since this study demonstrates that OVs can effectively deliver siRNA payloads that impact replication through immune-related pathways, there is a clear opportunity to use a similar approach to define genes that can increase tumour cell immunogenicity and other parameters that have a core impact on OV-induced immune responses.

Pairing an OV with a chemotherapeutic compound can enhance anti-tumour efficacy beyond that observed with either agent alone [60,61,62]. Following single agent or combination therapy with oncolytic reovirus and/or cisplatin, transcriptional profiling of B16.F10 melanoma cells revealed that cisplatin alone was predominantly associated with changes in apoptotic pathways, while reovirus alone was associated with enhanced pro-inflammatory, pro-apoptotic, MHC class I and oncogene signalling [63]. Yet when both therapies were combined, the reovirus-induced changes in pro-inflammatory and immune pathways were almost completely ablated. This included a loss of activity across a range of Th1 and Th17 cytokines, MIP-1α and RANTES. Interestingly, cisplatin represents a chemotherapeutic agent that is incapable of inducing immunogenic cell death (ICD) [64], while reovirus requires activated anti-tumour immune responses for efficacy [65]. ICD is widely considered a prerequisite for generating functional T cells against tumour-associated targets [64,66]. Indeed, absence of one of the four key markers of ICD—namely, ecto-calreticulin, ATP, HMGB1 and type I IFNs—correlates with a reduction, or complete loss, of immunogenicity following tumour cell death [67,68]. This suggests that, despite the clear synergy achieved between reovirus and cisplatin therapy [63], this combination may not be optimal if the focus is to achieve an immunostimulatory outcome.

## 6. Using Large-Scale Approaches to Analyse Tumour Adaptations to Immunotherapy and Signatures of Success

Tracking the immune-related changes that occur in tumour tissue, and relating this to resident and/or infiltrating immune populations, can offer insights into the biological mechanisms that determine whether a tumour-bearing individual will have a positive or negative response to immunotherapy. Currently, these changes are poorly understood. Comparing differences in responding *vs.* non-responding tumours can offer insights into tumour adaptations, and uncover key differences behind the biological networks that drive tumours to remain unresponsive, or respond with stabilised disease, temporary regression or complete durable regression. Ultimately, such screens can uncover signatures of therapeutic success in the tumour microenvironment.

Lesterhuis *et al.* compared the genomic profile of regressing and non-regressing tumours following anti-CTLA4 immunotherapy in a dichotomously-responding mesothelioma model, where approximately 20% of mice responded to therapy [69]. Using weighted gene co-expression network analysis, 8 modules were identified that were active across all tumours. Two of these modules were significantly associated with a positive response to anti-CTLA4 treatment. An immune-related module was upregulated in regressing tumours, and was enriched for pathways such as granulocyte adhesion and diapedesis, and signalling through CD28, iCOS/iCOSL, TCR and PKC. A cancer-related module was downregulated in regressing tumours, and was enriched for genes involved in cell cycle regulation, cholesterol biosynthesis, and signalling during cancer development. Such insights can guide the design of new strategies to broaden immunotherapy response rates across larger patient populations by targeting hub genes that can disrupt an entire network. Indeed, following anti-CTLA4 therapy in the mesothelioma model, NOS2 activity was identified as an important hub for efficacy. Treating animals with a drug to enhance NO production during anti-CTLA4 therapy resulted in an improvement in treatment efficacy from 10% to 80% [69].

Khandelwal *et al.* performed a high-throughput co-culture siRNA screen to capture and identify ligands expressed on tumour cells that were capable of inhibiting cytotoxic T lymphocyte (CTL)-mediated killing [70]. Survivin-positive MCF7 breast cancer cells stably tagged with luciferase were incubated with siRNAs targeting 520 genes coding for transmembrane and cell surface proteins, followed by co-culture with anti-survivin CTL. Specific tumour cell death was measured as a reduction in luciferase signal. In addition to expected immunoregulatory hits, such as *CD274* (PD-L1) and *LGALS3* (Galectin-3), knockdown of C-C chemokine receptor type 9 (*CCR9*), GLI pathogenesis-related 1 like 1 (*GLIPR1L1*), growth hormone secretagogue receptor (*GHSR*) and carcinoembryonic antigen-related cell adhesion molecule 6 (*CEACAM6*) enhanced tumour cell lysis across multiple malignant cell lines. In extended analyses, knockdown of *CCR9* in patient HLA-matched tumour-infiltrating lymphocytes also increased killing in primary malignant pancreatic or melanoma cells [70]. This suggested that the normal function of CCR9 was profoundly immunosuppressive, which was further supported when the overexpression of CCR9 on tumour cells inhibited CTL-mediated lysis and enabled immune escape. To further elucidate the mechanism behind this inhibition, microarray analysis was performed on survivin-specific CTL encountering either CCR9^lo^ or CCR9^hi^ tumour cells. After an encounter with CCR9^lo^ tumour cells, genes involved in the positive regulation of the immune response, such as lymphotoxin alpha (*LTA*) and integrin alpha-2 (*ITGA2*), were over-expressed in CTL. Conversely, these same cells downregulated genes involved in lymphocyte maturation and apoptosis, such as ephrin-A1 (*EFNA1*) and inhibitor of DNA binding-1 (*ID1*). Thus, knockdown of *CCR9* favoured T cell activation, proliferation and survival.

Similarly, McGray *et al.* analysed changes in tumour transcriptomes at the tipping point where tumour-bearing animals began to respond to therapeutic cancer vaccination [40]. In this study, replication-incompetent rHuAd5 vectors expressing the melanoma antigen, human dopachrome tautomerase (hDCT), generated modest frequencies of hDCT-specific CD8+ T cells (~3%) in B16.F10 tumour-bearing animals to achieve a significant survival extension, but no durable cures. Tumour transcriptional analysis demonstrated that, as a rapid and early consequence of vaccination, several profoundly immunosuppressive genes were highly upregulated, including *CD274* (PD-L1), *LGALS9* (Galectin-9), *TGFβ1*, *LAG3* and *HAVCR2* (TIM-3). Further microarray analysis demonstrated the activation of pathways involved in antigen processing and presentation. Thus, vaccination resulted in profoundly immunosuppressive immune and non-immune changes that attenuated T cell activity and enabled continued tumour growth despite a continued expansion of T cell frequencies [40]. Although these suppressive mechanisms were still active—and even amplified—when large numbers of hDCT-specific CD8+ T cells were adoptively transferred in combination with rHuAd5 vaccination, the sheer magnitude of infused cells, which achieved wide distribution across the tumour, nevertheless led to durable cures in ~65% of tumour-bearing animals [40].

The ability to identify immune or viral markers of success following OVIT is important for predicting immune engagement and patient outcome. Zloza *et al.* performed microarray analysis to uncover changes in both T cells and cancer cells that reflected the level of immunotherapeutic engagement in metastatic melanoma patients treated with multiple intratumoural injections of oncolytic vaccinia virus (rVV-B7.1). Two responder and three non-responder patients were analysed. In bulk cells isolated from the tumour microenvironment, 55 upregulated and 37 downregulated genes were identified across all patients following treatment. Extended analysis demonstrated that one of these genes, *LILRB1* (ILT2), a member of the leucocyte immunoglobulin-like family that functions as an immunoregulatory receptor, was downregulated in responding patients, but was upregulated in non-responders. This suggested a potential correlation between the expression of this marker on immune cells within the tumour microenvironment and patient outcome. In extended analyses, melanoma patients who responded well to rVV-B7.1 had a lower frequency of ILT2-expressing FoxP3+ CD4+ and CD8+ T cells both in the blood and the tumour [71]. Thus, low expression of ILT2 may have value as a predictive biomarker for patients likely to respond to rVV-B7.1 therapy.

The pathways that tumours recruit to achieve immune escape following OV infection can similarly be captured by transcriptomic analysis. Tumours like 4T1 (murine breast carcinoma) and SKOV (human ovarian carcinoma) can develop OV resistance by re-establishing interferon expression (possibly indirectly, through stromal IFN stimulation) and thus rendering the malignant environment refractory to the replication of some OVs [72,73]. Other OVs, such as VV and HSV, are less sensitive to IFN reactivation [74]. Microarray analysis has captured several additional pathways through which other cancer cell lines, such as PLC5 (human hepatoma) and HT29 (human colorectal adenocarcinoma), can develop resistance. For example, following infection with HSV (strain G207), gene expression changes associated with resistance acted to decrease the availability of virally-accessed cell surface receptors, modify the stage of the cell cycle and reduce nucleotide pool sizes [75]. Similarly, by comparing the transcriptomes of cancer cell lines that were either resistant or sensitive to VSV∆51-induced oncolysis, pathways involved in endocytosis and IFN signalling were identified as molecular signatures of therapeutic resistance [76]. Finally, by analysing genes that were differentially regulated in the presence of virus alone, drug alone or combination therapy in drug- and VSV-resistant chronic lymphocytic leukaemia (CLL) cells treated with obatoclax, a Bcl-2 inhibitor drug under clinical development, two core pathways involving apoptosis signalling and mTOR signalling were required to achieve efficacy following combination therapy. In the combination therapy group, anti-apoptotic genes, such as *BIRC6*, were downregulated, while pro-apoptotic genes, such as *BID* and *DIABLO*, were upregulated [77]. Analysing the functional status of such pathways in patient tumour biopsies prior to OV treatment may represent a useful approach for predicting responses.

## 7. Future Directions

Improving the ability to easily and meaningfully integrate and interpret large-scale immune data generated from disparate biological sources will be key to improving the ability to achieve novel, therapeutically relevant insights for OVIT. Currently, the ability to integrate extremely complex data derived from multiple sources to generate actionable insights is a particular challenge in the field; yet several tools have been (and continue to be) designed to facilitate such analyses [18,78,79,80].

While the greatest volume of large-scale data has thus far been generated from analyses of bulk immune populations, additional layers of complexity will be generated from single cell approaches. For example, Lu *et al.* recently reported the ability to co-detect 42 immune effector proteins secreted from a single cell following exposure to an immune stimulus using a multiplex microchip assay [81]. This approach achieved two key insights: firstly, even a phenotypically pure immune cell population displayed substantial heterogeneity at the single cell level in terms of activation and functional capacity. Secondly, only a subset of this phenotypically pure population was capable of potentiating cytokine activation following immune activation. Such insights, and the ability to perform comprehensive immune monitoring at the individual cell level, will have broad utility for OVIT.

In the mouse, the use of high-dimensional techniques to track myeloid populations in lymphoid and non-lymphoid tissues by mass cytometry and cluster analysis has already begun to allow the more rigorous and unambiguous definition of immune populations. Using this technique, Becher *et al.* used a 38 antibody panel to catalogue considerable variation in neutrophil phenotypes, yet a remarkably consistent phenotype for other immune populations, such as plasmacytoid DCs, across tissues [82]. While unambiguous definition of certain immune populations required the incorporation of additional antibodies, cellular differentiation and activation states could easily be tracked as a series of progressive phenotypic changes [82]. This approach has so far only been performed in the myeloid compartment of mice at steady state. However, extending this powerful technology to incorporate lymphoid compartments, and to subsequently explore responses to infection, vaccination and immunotherapy, will greatly enhance our capacity to achieve broad insights. The implementation of new large-scale assays to assess the contribution of regulatory elements, such as chromatin profiling [83], will also be important tools to study more complex levels of immune regulation during OVIT.

Finally, large-scale technologies have clear potential to provide guidance for addressing several key issues that currently hamper the development of successful immune responses during OVIT, such as immune cell recruitment, antigen priming and cross-presentation to drive broader immune responses against multiple tumour antigens that are released as oncolysis proceeds, and immune suppression at the tumour site. Improving accessibility to and uptake of large-scale technologies will facilitate the exploration of these critical areas of research, which have the potential to extend the benefits of OVIT across wider patient populations.

## 8. Final Thoughts

Enhancing the breadth and depth of our knowledge about the major and minor contributions of specific and global immune cell populations during OVIT is critical to inform the design of new approaches to strengthen therapeutic outcomes. Large-scale assays can interrogate and integrate the genome, transcriptome, proteome and metabolome, among others, in either an empirical or unbiased way to generate data-rich insights. While the bulk of the large-scale immune-related data generated thus far has focussed on either infectious models from a public health perspective or therapeutic models using non-oncolytic immunotherapies, there is clear untapped value and a strong rationale for applying these current and emerging approaches in the OVIT setting.
